# Supplementary material for: Rapid Growth between 0 and 2 Years Old in Healthy Infants Born at Term and Its Relationship with Later Obesity: A Systematic Review and Meta-Analysis of Evidence
Source: Nutrients. 2024 Sep 2;16(17):2939. doi: 10.3390/nu16172939 (PMC11397548; doi:10.3390/nu16172939)
Supplement: Supplementary file 1 [file nutrients-16-02939-s001.zip › nutrients-3125901-supplementary.pdf]

Forest plots corresponding to the meta-analysis performed afterexcluding th e studies with the highest sample weight.

Figure S1. Forest plot of the analysis of the mean difference in standard deviation of height after excluding the study with the highest sample weight (Akaboshi et al. [45]).

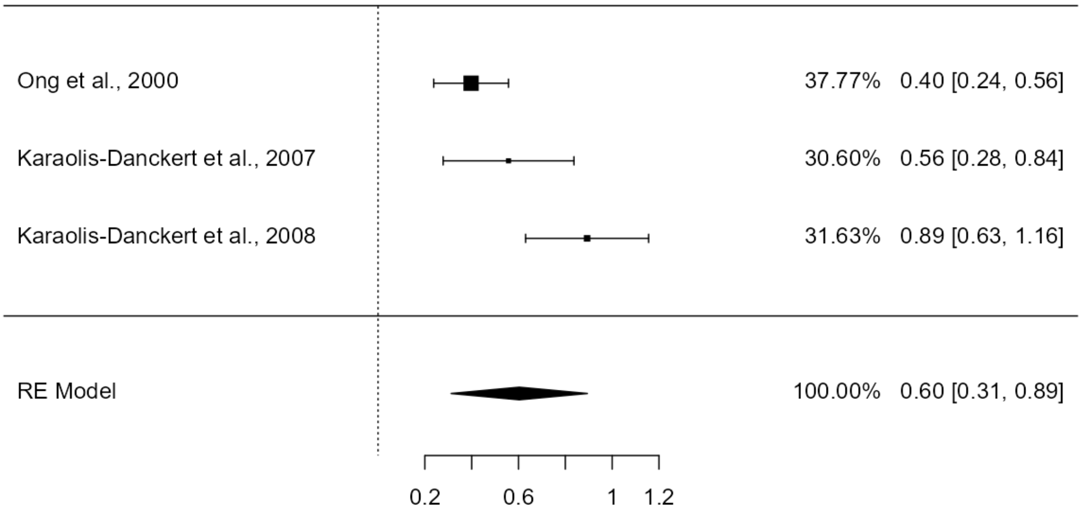

Note: RE: random effect.

Figure S2. Forest plot of the analysis of the mean difference in standard deviation of weight after excluding the study with the highest sample weight (Akaboshi et al. [45]).

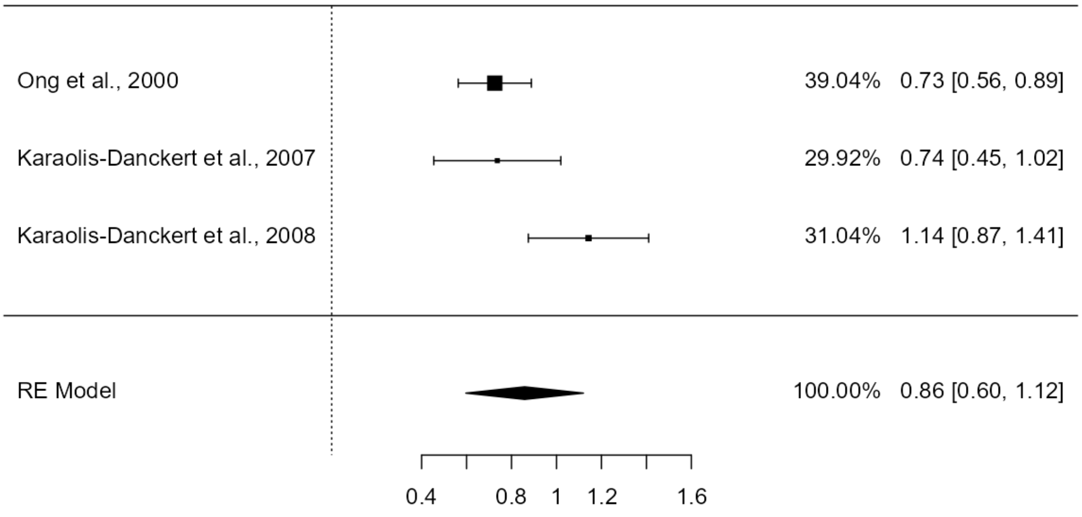

Note: RE: random effect.

Figure S3. Forest plot of the analysis of the mean difference in standard deviation of BMI after excluding the study with the highest sample weight (Ong et al. [39]).

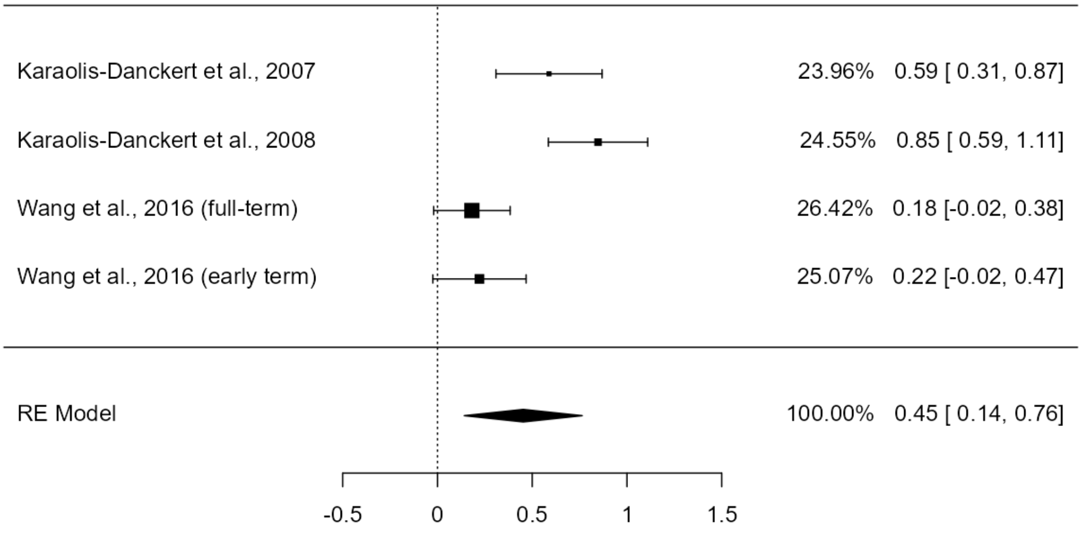

Note: RE: random effect.

Figure S4. Forest plot of the analysis of the difference in logarithmic odds ratios after excluding the study with the highest sample weight (Wang et al. [42]).

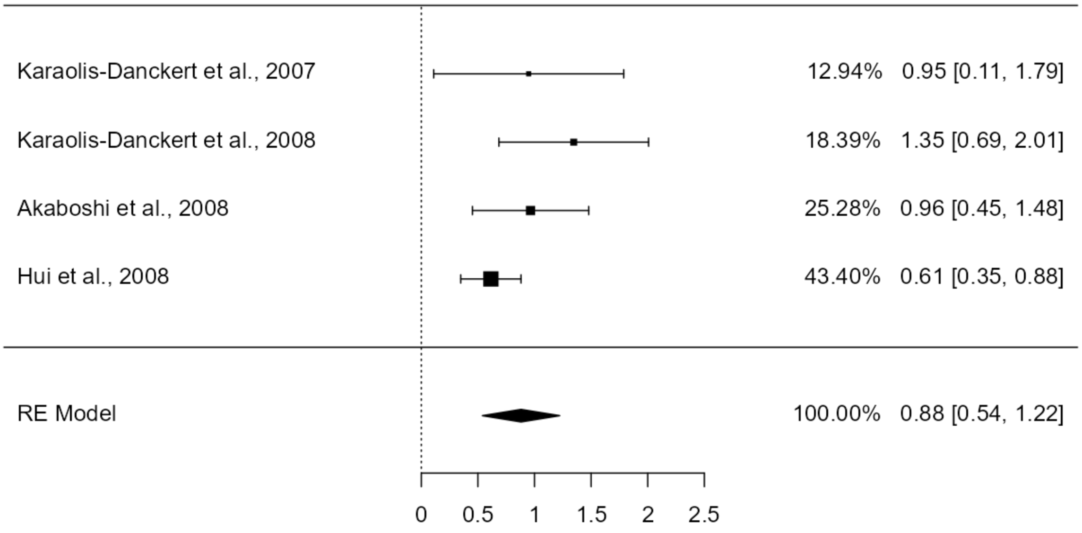

Note: RE: random effect.
